# IgG4-Related Disease With Gastrointestinal Involvement: Case Reports and Literature Review

**DOI:** 10.3389/fimmu.2022.816830

**Published:** 2022-03-10

**Authors:** Xinhe Zhang, Xing Jin, Lin Guan, Xuyong Lin, Xuedan Li, Yiling Li

**Affiliations:** ^1^Gastroenterology Department, The First Affiliated Hospital of China Medical University, Shenyang, China; ^2^Department of Pathology, The First Affiliated Hospital of China Medical University, Shenyang, China; ^3^Radiology Department, The First Affiliated Hospital of China Medical University, Shenyang, China

**Keywords:** IgG4-related disease, IgG4-related gastric disease, IgG4-related sclerosing cholangitis, atrophic gastritis, autoimmune diseases

## Abstract

IgG4-related disease is an immune-mediated chronic, systemic, and autoinflammatory disease that can affect various organs throughout the body. The most commonly affected areas are the pancreas and biliary system. Due to the diverse clinical manifestations of the disease, it affects widely distributed organs. Thus, it is often easy to misdiagnose or miss. The digestive tract is a rarely affected system, and most IgG4-related gastric diseases manifest as tumors detected by endoscopy. This article reports two special cases with IgG4-related disease involving atrophic gastritis and intestinal polyps to provide a more empirical and theoretical basis for clinical diagnosis and treatment.

## Introduction

IgG4-related disease (IgG4-RD) is a chronic, systemic, and autoinflammatory disease mediated by the immune system. Although this disease may be classed as ‘autoinflammatory’, in fact this disease has many autoimmune manifestations, such as jaundice, fatigue, joint pain, enlarged glands, etc. The main clinical features of the disease are swelling, fibrosis, and sclerosis of the affected organs. The concentration of serum IgG4 is significantly increased. The affected tissues and organs are infiltrated by a large number of lymphocytes that form germinal centers, in particular, IgG4-positive plasma cells. The disease may involve various organs, such as the pancreas, biliary gland, lymph node, eye, thyroid, lung, kidney, prostate and skin (1). The biliary gland is one of the most commonly involved organs in IgG4-RD. However, it is rare that IgG4-positive cells infiltrate the digestive tract, and most cases of IgG4-related gastric disease show tumors under endoscopy and are diagnosed after surgery. Therefore, it is more difficult to identify in patients who show gastritis under endoscopy. This study reports the cases of two patients with IgG4-RD with a space-occupying lesion of the bile duct. The patients were ultimately diagnosed with IgG4-RD with digestive tract involvement by multiple diagnostic strategies. We review the related diagnosis and treatment to provide more information for clinical practice.

## Case 1

**Figure 1:**
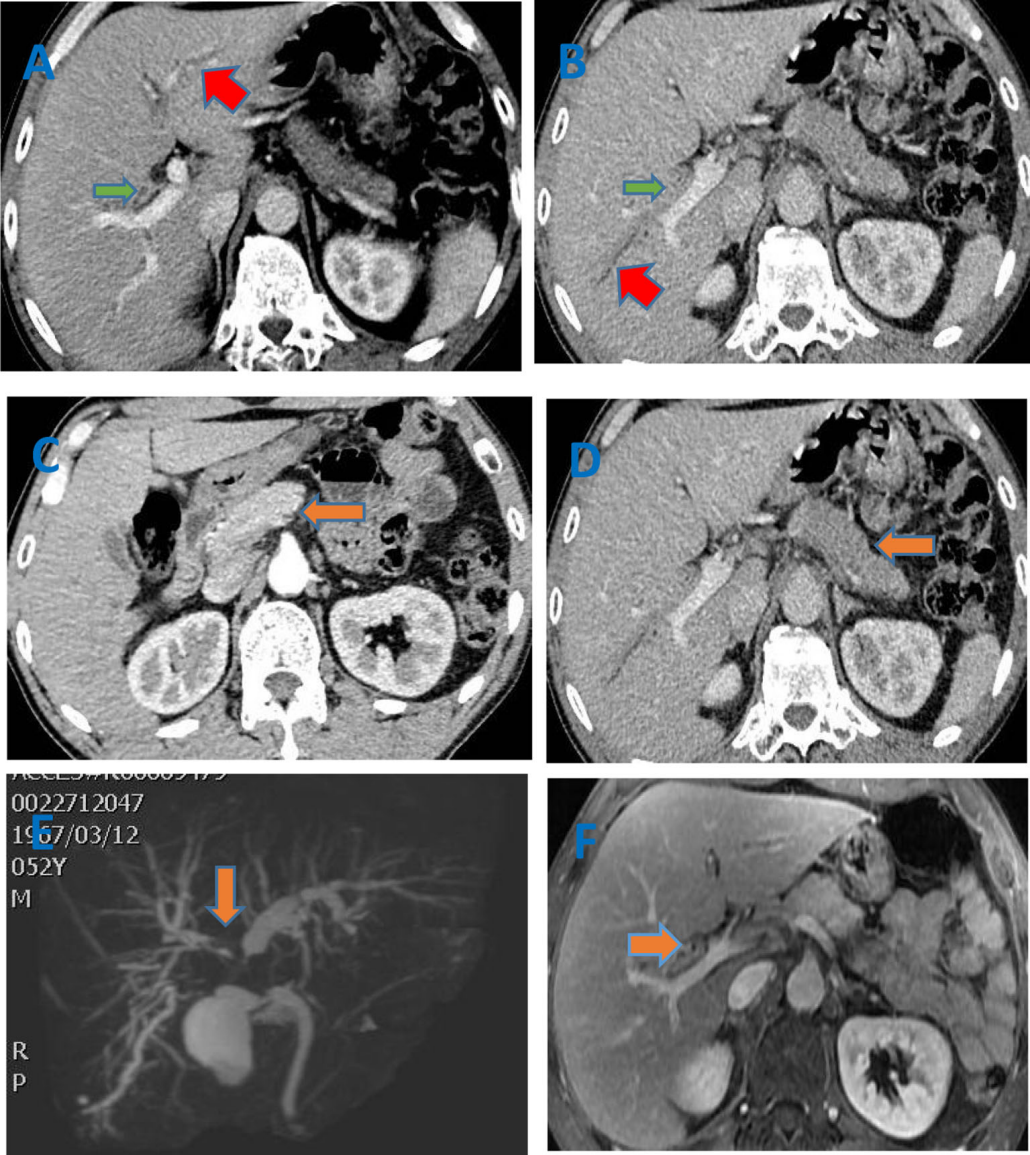
**(A–D)** Enhanced CT of the abdomen showed that the common hepatic duct, the beginning of the left hepatic duct (red arrow), the right hepatic duct (green arrow) and its branch walls were thickened and gradually strengthened. The intrahepatic bile ducts were slightly dilated. The pancreas (orange arrow) was full, and the edges were irregular. After the enhancement, the enhancement at the arterial phase decreased and showed low density. But it further enhanced with the delay. No pancreatic duct dilation was seen; **(E)** Biliary MRCP indicated multi-segment lumen stenosis of the bile duct. The thickening of the tube wall involved a large extent, which did not match the degree of lumen expansion. The thickening of the tube wall was obvious, irregular, and asymmetric; **(F)** The enhanced MRI of liver indicated that the bile duct wall was gradually strengthened. The pancreas was full with straight contour. The T2 signal was increased,and the TI signal was reduced. The enhancement was uniform. The pancreatic duct was not dilated.

**Figure 2:**
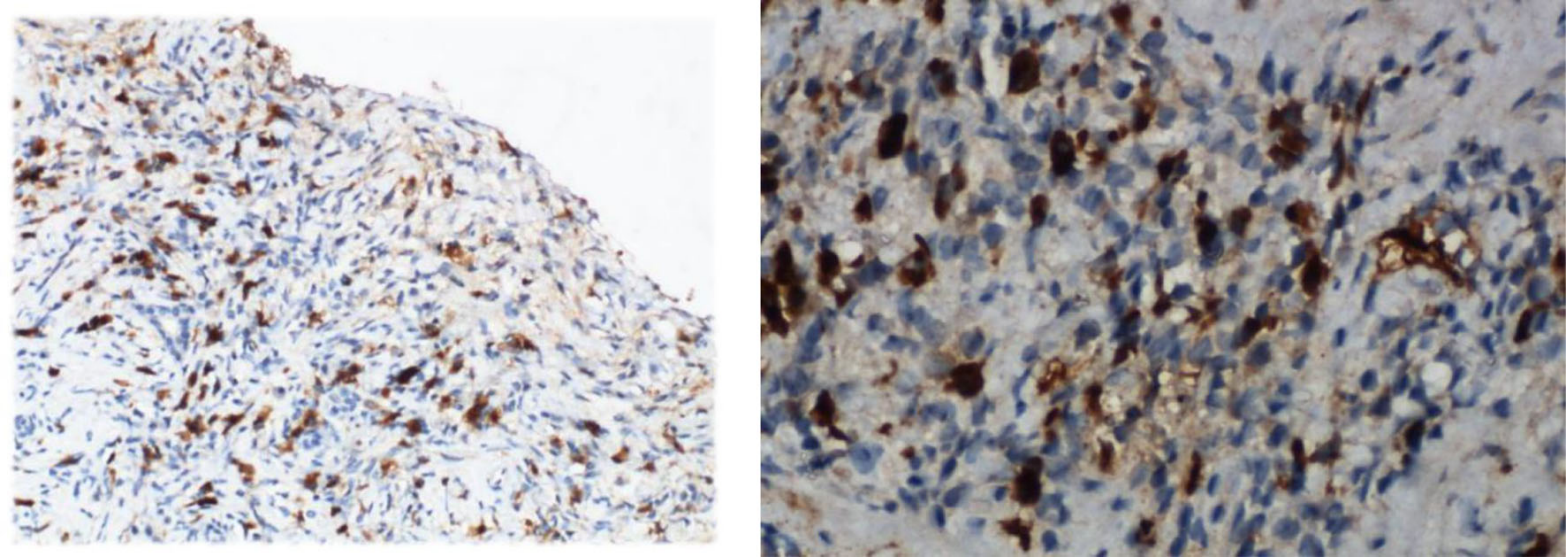
The immunohistochemical staining for IgG4 of the left submandibular gland and gastric antrum mucosal tissue (Case 1).

A 52-year-old male presented with yellow urine and abdominal distension for more than one month and yellowish skin and sclera for 20 days. There was no obvious cause for the darkening of the urine, visible abdominal distension or aversion to oily foods. However, the patient was not concerned about these symptoms. After ten days, he developed yellowish skin and sclera. However, there was no fever, fatigue, skin itching, nausea, vomiting, abdominal pain, or significant change in body weight. Therefore, the patient went to a local hospital for a test for abnormal liver function indicators. Image indicated obstruction at the hilar biliary and hilar occupation at the bile duct. Accordingly, hilar cholangiocarcinoma was likely. After admission, relevant laboratory examinations were performed. Liver function test including AST, ALT, ƴGT, ALP, total bilirubin were all elevated. The autoimmune antigen spectra were all negative. The IgG4 subtype test revealed an IgG4 level of 5.22 g/L (normal value 0.03-2.01 g/L), which was significantly higher than the upper limit of normal. Infection-related indicators (C-reactive protein and procalcitonin) and tumor markers had no abnormalities. Other tests (Full blood cound basic biochemistry test, thyroid function test, coangulation test, serum protein electrophoresis, immynoglobulin levels) were all normal. Doppler ultrasound of the liver, gallbladder and spleen showed that the inner diameters of the left hepatic duct, right hepatic duct, and common bile duct were approximately 0.72 cm, 0.86 cm, and 1.06 cm, respectively. This result indicated that the bile ducts inside and outside of the liver were dilated. The common bile duct was not clearly displayed, and hypoechoicity could be seen in partial areas. Space-occupying lesions could not be excluded. The patient also completed computed tomography (CT), magnetic resonance cholangiopancreatography (MRCP) and magnetic resonance imaging (MRI) of the abdomen (**Figure 1**). Color Doppler ultrasound of the submandibular gland showed several hypoechoic echoes in the bilateral submandibular glands. A 2.82×1.18 cm hypoechoic echo was seen next to the left submandibular gland. Color Doppler ultrasound of the superficial lymph nodes showed grade 4 swelling of the bilateral lymph nodes of the neck and supraclavicular fossa. Grade 2 swollen lymph nodes were observed in the bilateral axillary and inguinal areas. Gastroscopy revealed that the mucosa of the gastric antrum was thinning and uneven. This indicated atrophic gastritis. The size and shape of the duodenal papilla were normal. We performed a needle biopsy of the left submandibular gland, left submandibular lymph node and gastric antrum mucosal tissue. The pathological diagnosis showed (**Figure 2**) focal fibrous tissue of the left submandibular gland hyperplasia and lymphocyte infiltration. Lymphocytes and plasma cells infiltrated the submucosa of the gastric antrum. The results of the immunohistochemical analysis were as follows: the left submandibular gland cells were positive for IgG4 (+75/High Power Field (HPF)), CK (epithelial +), CD3 (+), CD20 (+), Ki-67 (5%+); the left submandibular lymph node cells were positive for IgG4 (+25/HPF), CK (epithelial +), CD3 (interregion +), CD20 (follicle +), Ki-67 (5% +); and the gastric antral mucosal tissue was positive for IgG4 (+75/HPF), CK (epithelial+), CD3 (+), CD20(+), CD138(+), Ki-67(10%+). For atrophic gastritis the patient had test performed for H pylori. C13 urea breath tests and urease tests of gastric mucosal tissue were negative. No H.pylori was detected by endoscopic histology. The patient was ultimately diagnosed with IgG4-related diseases (IgG4-related pancreatitis, IgG4-related sialadenitis, IgG4-related sclerosing cholangitis, and IgG4-related gastric disease). The patient was given methylprednisolone 40 mg intravenously once a day. After 3 days of intravenous administration, the patient was given oral metoprolol 32 mg instead. The dose was reduced by one tablet per week. The final dosage was maintained at 4 mg. Simultaneously, silibinin capsules were prescribed for liver protection. Symptomatic acid suppression and calcitriol supplementation were needed. The patient follow-up continued. After 3 months of taking the drug, the abdominal distension, yellow urine, and yellowish skin and sclera were all relieved. There were no adverse reactions, such as fever or electrolyte imbalance. IgG4 levels had dropped to 0.88g/L, within the normal range. Although gastroscopy still showed atrophic gastritis, immunohistochemistry of the gastric mucosal tissue biopsy was negative for IgG4.

## Case 2

**Figure 3:**
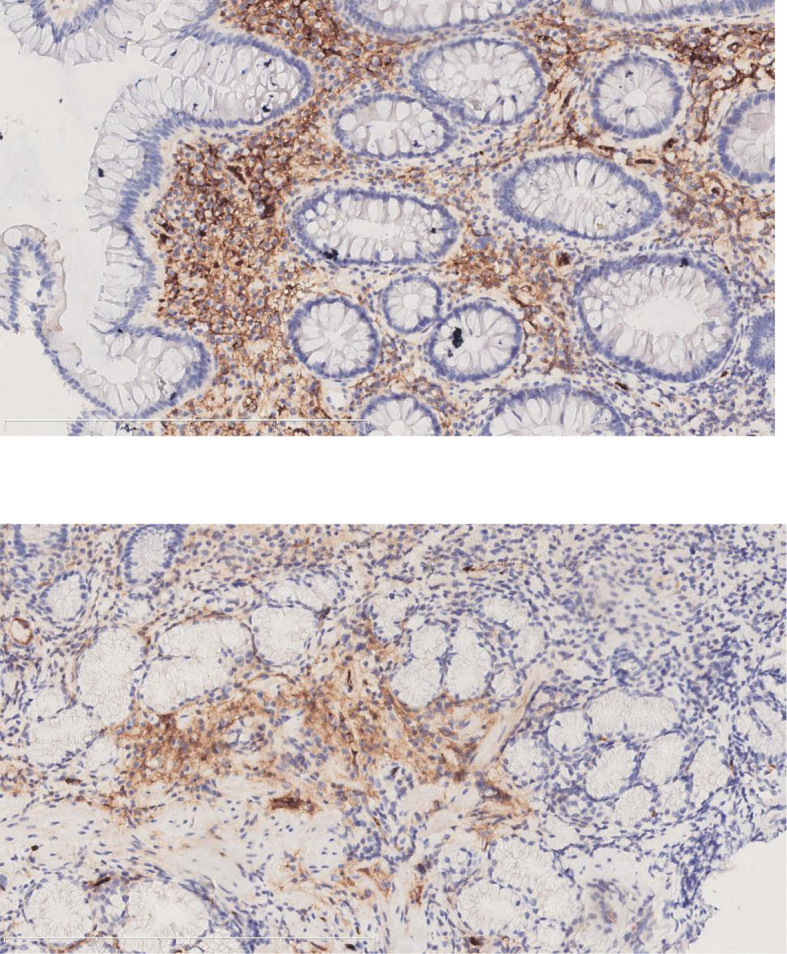
The immunohistochemical staining for IgG4 of the submandibular gland and intestinal polyps (Case 2).

A 65-year-old male presented with fatigue and edema of both lower extremities for three months. One month ago, the patient felt his fatigue was aggravated and accompanied by darkening of urine. Liver function test including AST, ALT, ƴGT, ALP, total bilirubin were all elevated. The IgG4 subtype test revealed an IgG4 level of 7.69 g/L. The results of lymph node ultrasound showed that several lymph nodes with a size of 1-2cm were seen on both sides of the neck, supraclavicular fossa and submandibular gland. Parotid glands and lacrimal glands on both sides enlarged, and echoes were uneven reduced. Enhanced CT of the abdomen showed gallstone and thick colon wall with stratified strengthening. Therefore, the patient underwent gastrointestinal endoscopy. Endoscopy revealed chronic atrophic gastritis and multiple colon polyps. We took pathology for the submandibular gland, stomach tissue and intestinal polyps. The pathological diagnosis showed (**Figure 3**) the infiltration of IgG4 cells. The results of the immunohistochemical analysis were as follows: the submandibular gland cells were positive for IgG4 (40/HPF), CD38 (+); the stomach tissue were positive for IgG4 (15/HPF), CK (epithelial +), CD38 (interregion +), CEA (+), Ki-67 (80% +); and intestinal polyps was positive for IgG4 (+40/HPF), CD38 (interregion +), Ki-67(95%+). The patient was ultimately diagnosed with IgG4-related gastrointestinal diseases. After the patient was admitted to the hospital, he received drugs to protect the liver and reduce jaundice, such as ursodeoxycholic acid and bicyclol. Under more than one month of treatment, the symptoms improved significantly and the liver function indicators gradually get better. The follow-up is currently ongoing at present.

## Discussion

Serum IgG4 elevation can occur in many diseases, such as IgG4-RD, autoimmune liver disease, tumors, and viral hepatitis. IgG4-RD is the most common cause of serum IgG4 elevation. Studies have shown that the sensitivity of serum IgG4>135 mg/dL for the diagnosis of IgG4-RD is 97.0%, and the specificity is 79.6%. IgG4≥2.8 g/L can help distinguish IgG4-RD from non-IgG4-RD diseases and evaluate the degree of organ involvement and the risk of recurrence (2). However, other diseases can also lead to an increase in IgG4. The study of Yang showed that 22.2% of non-IgG4-RD patients had rheumatic diseases with elevated IgG4 (3). And the elevation of IgG4 can be seen in allergy, tumor, and infection-related diseases (4)

The role of IgG4 in the immune system is reflected in many aspects. First, due to the unique structural characteristics of IgG4, the heterogeneity of the Fab segment leads to less flexibility and the inability to form immune complexes (5). Secondly, the Fc segment of IgG4 can not activate of complement C1q (6), which belongs to low complement affinity. It cannot activate the classical complement pathway and inflammatory cells to clear the antigen. So it has certain anti-inflammatory protective effect. Secondly, IgG4 promotes the occurrence and development of malignant tumors through immune tolerance and immune escape mechanisms (7). Although IgG4 seems to have protective effect, it is still pathogenic. IgG4 levels are elevated in some immune diseases and positively correlated with severity. But whether IgG4 is pathogenic in IgG4-RD should be further explored. Th2 immune response plays an important role in IgG4-RD. Th2 cytokines (IL-4, IL-10, IL-13) are highly expressed in IgG4-RD damaged tissue and peripheral monocytes. IL-4 and IL -13 have been shown to promote the conversion of IgG1 to IgG4 (8). This process requires the recognition of T and B cells. So the cytokines of CD4+ T cells and follicular helper T cells (Tfhs) also play an important role in IgG4-RD.

Akiyama (9) found that the increase in serum IgG4 levels is related to the Tfh2 subtype of Tfhs. Tfh2 cells can induce naive B cells to differentiate into plasma cells and produce IgG4. Interleukin 4 (IL-4) and interleukin 21 (IL-21) secreted by Tfh2 cells are involved in this process. Mattoo (10) found that the content of cytotoxic CD4+ T cells in the peripheral blood of patients with IgG4-RD was significantly increased and directly proportional to the level of IgG4. CD4+ cytotoxic T lymphocytes (CTL) express SLAMF7, perforin, granzyme, IL-1, TGF, and interferon γ, which may be important contributors to chronic inflammation and fibrosis in IgG4-RD pathology. The histopathological diagnosis of IgG4-RD includes dense lymphoplasmacytic infiltration, fibrosis, and obliterative phlebitis. Therefore, cytokines involved in the elevation of IgG4 are important to the pathological changes.

Regulatory T cells (Treg) cells in IgG4-RD lesions produce IL-10 and TGF-β. IL-10 is associated with antibody switching of B cells, and TGF-β is associated with tissue fibrosis (11). In addition, more than half of IgG4-RD patients show hypocomplementemia, which may be related to IgG4. Anti-F(ab’)2 antibody, also known as anti-hinge antibody (AHA), is rheumatoid arthritis-specific antibody. IgG4 is decomposed into F(ab)’2 fragments. F(ab)’2 and AHA immune complexes can activate complement, resulting in low complement blood (12)

After the diagnosis, we identified the patient’s affected organs. One of the most commonly involved organs in IgG4-RD is the pancreas, and the resulting disease is referred to as IgG4-related pancreatitis. The diagnosis is mainly based on typical imaging findings and laboratory examinations. Imaging examination of the patient revealed fullness and swelling of the pancreas, and this finding combined with the IgG4 indicators resulted in the diagnosis of IgG4-related pancreatitis (13). Bile duct involvement in IgG4-RD is referred to as IgG4-related sclerosing cholangitis (IgG4-RSC). IgG4-RD has no specific clinical manifestations and is often associated with autoimmune pancreatitis. It can be diagnosed if there is a high serum IgG4 concentration in combination with bile duct stenosis on imaging examinations. IgG4-RSC is divided into 4 types according to the location of the bile duct stricture as follows: type 1: lower common bile duct stenosis; type 2: diffuse stenosis of the intrahepatic and extrahepatic bile ducts; type 3: hilar bile duct and lower common bile duct stenosis; and type 4: bile duct stenosis in the hilar area (14). According to the imaging findings of this patient, this patient had type 4 IgG4-RSC. The most challenging aspect of diagnosing this patient was distinguishing between IgG4-RSC and cholangiocarcinoma. However, there are still significant differences between IgG4-RSC and cholangiocarcinoma. In terms of clinical manifestation, patients with gallbladder cancer may have steatorrhea and abdominal pain. This is rarely reported in patients with IgG4-RSC (15). Obvious elevation of tumor markers has great value for the diagnosis of cholangiocarcinoma. Because the clinical manifestations, imaging manifestations and pathology findings of this patient were difficult to obtain, we did not initially exclude the possibility of both IgG4-RSC and cholangiocarcinoma being present. A conservative medication strategy was chosen (meprednisone, silibinin capsules, omeprazole, calcitriol). Fortunately, after this patient was treated with hormones, his condition improved significantly, proving that hormone therapy was effective for this patient. This allowed us to rule out the diagnosis of cholangiocarcinoma.

To our surprise, we found that the patients had gastrointestinal involvement, which is less common in IgG4 disease and is easily missed. At present, there are few reported cases of IgG4-RDs involving the digestive tract. A meta-analysis suggests that the relative risk for IgG4-RD and gastric cancer can reach 1.69 (16). Skorus U reviewed cases of IgG4-related diseases involving the stomach. In this systematic review, only 3 of the 9 patients presented with gastrointestinal discomfort, and the lesions were found accidentally in the other patients. Most of the patients had submucosal tumors, and only one had gastric ulcers. The latest literature summarized 39 IgG4-RD patients with gastroesophageal involvement, and only 40% of the patients had typical gastric manifestations. Most of the lesions in patients with gastric involvement were inflammatory tumors, ulcers, nodular lesions, areas of chronic gastritis, and malignant lesions (17).

**Table 1** shows the cases of IgG4-related gastric disease reported in articles available from PUBMED (18–28). Most patients had tumors and were diagnosed by pathology after surgery, and the disease may be related to suppressor T cells. Della-Torre hypothesized that the pathogenesis of IgG4-RD resulted from the effect of T cells and B cells on certain specific antigens (8). A long-term chronic immune response is induced, and suppressor T cells provide an environment for inflammatory factors in this immune response. Suppressor T cells also play an important role in the occurrence of malignant tumors. IgG4 is closely related to the occurrence and development of tumors through immune escape and escape tolerance. In the study of melanoma (29), IL-10 induces Th2-type immune response to stimulate the production of IgG4 and reduce the interferon and TNF, which reduces tumor cell phagocytosi and inhibits the antitumor immune response. In the study of esophageal cancer (30), IgG4 inhibited the classic immune reactions through its Fc fragment reacting to the Fc fragments of cancer-specific IgG1. IgG4 competed with IgG1 in reacting to Fc receptors of immune effector cells. Therefore, locally increased IgG4 help cancer to evade local immune attack and indirectly promote cancer growth. However, whether the specific mechanism is also applicable to the link between gastric cancer and IgG4, it needs to be further explored the possible mechanism in human cancer samples and animal tumor models through a large number of *in vitro* and *in vivo* techniques. Very few patients could be diagnosed with other gastric diseases by gastroscopy. Similar to the previous report, the two cases were diagnosed IgG4-RD involving the gastrointestinal tract with elevated IgG4, which were confirmed by endoscopic biopsy and IgG4 immunohistochemical staining. But it is different that our patients have been diagnosed with IgG4-RD through symptoms, tests, examinations, and pathology. During the process of exploring the related organs, routine endoscopic screening was used to confirm the involvement of the gastrointestinal tract. And the patients in our cases were diagnosed with atrophic gastritis and multiple colon polyps by endoscopy, which is a special and amazing discovery among IgG4-RD patients. Furthermore, IgG4-RD can not only affect the stomach but can also be limited to the stomach. Patients with multiple organ IgG4-RD are mainly elderly men and often have elevated serum IgG4 levels. In contrast, isolated gastric IgG4-RD mainly affects female patients with normal serum IgG4. However, the difference between the two disease presentations needs to be further studied and determined. It is important to note that the concentration of serum IgG4 is only measured in a small number of patients before surgery. Therefore, the stomach is often ignored in IgG4-RD.

**Table 1:** Cases of IgG4-related gastric disease.

Study	Age at diagnosis	Gender	Serum IgG4 (g/L)	Number of IgG4, ratio of IgG4/IgG	Endoscopic finding	Kind of lension	Size	Other related organs	Treatment
Yamane T et al.	70	Female	Not check	210/Hpf, >80%	mass	Submucosal tumor	10mm	pancreas	Surgery (resection)
Probst A et al.	71	Female	Not check	98/HPF, 45%	Ulcer, thickening of the gastric wall	tumor	–	none	Surgery (resection)
Probst A et al.	76	female	Not check	50/HPF, 56%	Ulcer, thickening of the gastric wall	tumor	–	none	Surgery (resection)
Woo CG et al.	48	Female	Not check	210/Hpf, 85%	mass	subepithelial tumor	36*22mm	none	Surgery (resection)
Seo HS et al.	40	Female	Not check	-/20%-40%	mass	subepithelial tumor	43*27mm	none	Surgery (resection)
Inoue K et al.	74	Male	11.20	172/Hpf, 81.5%	Mass	early gastric cancer	15mm	kidney	Drug
Cho MJ et al.	45	Male	Not check	60/Hpf, 40%	mass	subepithelial tumor	30*30mm	None	Surgery (resection)
Berger Z et al.	71	Female	9.68	25-40/Hpf	Mucosal thickness	hypertrophic gastropathy	–	pancreas	Drug
Bulanov D et al.	62	Female	Not check	50/Hpf	ulcer	tumor	30*80mm	none	Surgery (resection)
Muto O et al.	26	Male	1.54	10/Hpf, 40%	ulcer	ulcer	–	none	Drug
Lim DY et al.	81	male	1.22	–	Ulcer	tumor	–	none	Drug
Bohlok A et al.	57	male	Not check	50/Hpf, 40%	mass	gastric antral lesion	17.7*16 mm	none	Surgery (resection)

## Conclusion

The incidence of IgG4-related diseases is gradually increasing. Its clinical manifestations are diverse, and the involved organs are widely distributed. Therefore, it is necessary to pay attention to the screening of multiple organs in clinical examinations. The possibility of IgG4-related diseases must be considered in addition to malignant tumors, especially in the presence of biliary space-occupying lesions.

## Data Availability Statement

The original contributions presented in the study are included in the article/supplementary material. Further inquiries can be directed to the corresponding author.

## Ethics Statement

The studies involving human participants were reviewed and approved by the First Affiliated Hospital of China Medical University. The patients provided written informed consent to participate in this study. Written informed consent was obtained from the individuals for the publication of any potentially identifiable images or data included in this article.

## Author Contributions

YL designed the study. XZ and XJ wrote the original draft. LG collected the case. XLin and XLi provided the photo. XZ and YL reviewed and edited. All authors contributed to the article and approved the submitted version.

## Conflict of Interest

The authors declare that the research was conducted in the absence of any commercial or financial relationships that could be construed as a potential conflict of interest.

## Publisher’s Note

All claims expressed in this article are solely those of the authors and do not necessarily represent those of their affiliated organizations, or those of the publisher, the editors and the reviewers. Any product that may be evaluated in this article, or claim that may be made by its manufacturer, is not guaranteed or endorsed by the publisher.

## References

[1] Sánchez-Oro R Alonso-Muñoz EM Martí Romero L. Review of IgG4-Related Disease. Gastroenterol Hepatol (2019) 42(10):638–47. doi: 10.1016/j.gastrohep.2019.08.009 31722794

[2] Pelkmans LG Hendriksz TR Westenend PJ Vermeer HJ van Bommel EFH. Elevated Serum IgG4 Levels in Diagnosis, Treatment Response, Organ Involvement, and Relapse in a Prospective IgG4-Related Disease UK Cohort. Clin Rheumatol (2017) 36(4):903–12. doi: 10.1007/s10067-017-3542-8 28105551

[3] Yang H Li J Wang Y Ye S Li J. Distribution Characteristics of Elevated Serum Immunoglobulin G 4 (IgG 4) and its Relationship With IgG 4-Related Disease. Scand J Rheumatol (2019) 48(6):497–504. doi: 10.1080/03009742.2019.1602882 31354076

[4] Hsieh SC Shen CY Liao HT Chen MH Wu CH Li KJ. The Cellular and Molecular Bases of Allergy, Inflammation and Tissue Fibrosis in Patients With IgG4-Related Disease. Int J Mol Sci (2020) 21(14):5082. doi: 10.3390/ijms21145082 PMC7404109 32708432

[5] Davies AM Sutton BJ. Human IgG4: A Structural Perspective. Immunol Rev (2015) 268(1):139–59. doi: 10.1111/imr.12349 PMC4670484 26497518

[6] Spiegelberg HL. Fc Receptors for IgE and Interleukin-4 Induced IgE and IgG4 Secretion. J Invest Dermatol (1990) 94(6):49S–52S. doi: 10.1111/1523-1747.ep12875051 2191055

[7] Bianchini R Karagiannis SN Jordakieva G Jensen-Jarolim E. The Role of IgG4 in the Fine Tuning of Tolerance in IgE-Mediated Allergy and Cancer. Int J Mol Sci (2020) 21(14):5017. doi: 10.3390/ijms21145017 PMC7404042 32708690

[8] Della-Torre E Lanzillotta M Doglioni C. Immunology of IgG4-Related Disease. Clin Exp Immunol (2015) 181(2):191–206. doi: 10.1111/cei.12641 25865251 PMC4516435

[9] Akiyama M Yasuoka H Yamaoka K Suzuki K Kaneko Y Kondo H. Enhanced IgG4 Production by Follicular Helper 2 T Cells and the Involvement of Follicular Helper 1 T Cells in the Pathogenesis of IgG4-Related Disease. Arthritis Res Ther (2016) 18:167. doi: 10.1186/s13075-016-1064-4 27411315 PMC4944254

[10] Mattoo H Mahajan VS Maehara T Deshpande V Della-Torre E Wallace ZS. Clonal Expansion of CD4(+) Cytotoxic T Lymphocytes in Patients With IgG4-Related Disease. J Allergy Clin Immunol (2016) 138(3):825–38. doi: 10.1016/j.jaci.2015.12.1330 PMC5014627 26971690

[11] Horii M Matsushita T. Roles of Regulatory T and B Cells in IgG4-Related Disease. J Mol Biol (2021) 433(1):166685. doi: 10.1016/j.jmb.2020.10.019 33096106

[12] Peng L Lu H Zhou J Zhang P Li J Liu Z. Clinical Characteristics and Outcome of IgG4-Related Disease With Hypocomplementemia: A Prospective Cohort Study. Arthritis Res Ther (2021) 23(1):102. doi: 10.1186/s13075-021-02481-3 33827676 PMC8025345

[13] Kawa S. Current Concepts and Diagnosis of IgG4-Related Pancreatitis (Type 1 AIP). Semin Liver Dis (2016) 36(3):257–73. doi: 10.1055/s-0036-1584318 27466795

[14] Kamisawa T Nakazawa T Tazuma S Zen Y Tanaka A Ohara H. Clinical Practice Guidelines for IgG4-Related Sclerosing Cholangitis. J Hepatobiliary Pancreat Sci (2019) 26(1):9–42. doi: 10.1002/jhbp.596 30575336 PMC6590186

[15] Zhang YA Shen XZ Zhu JM Liu TT. Extensive Metastatic Cholangiocarcinoma Associated With IgG4-Related Sclerosing Cholangitis Misdiagnosed as Isolated IgG4-Related Sclerosing Cholangitis. Med (Baltimore) (2015) 94(45):e2052. doi: 10.1097/MD.0000000000002052 PMC4912306 26559312

[16] Song M Latorre G Ivanovic-Zuvic D Camargo MC Rabkin CS. Autoimmune Diseases and Gastric Cancer Risk: A Systematic Review and Meta-Analysis. Cancer Res Treat (2019) 51(3):841–50. doi: 10.4143/crt.2019.151 PMC6639229 31048663

[17] Khan S Zhu LP Jiang K Liu W Chen X Wang BM. Immunoglobulin G4-Related Disease Manifesting as Isolated, Typical, and Nontypical Gastroesophageal Lesion: A Research of Literature Review. Digestion (2020) 101(5):506–21. doi: 10.1159/000501513 31291621

[18] Yamane T Eto K Morinaga T Matsumura K Yamashita K Tokunaga R. IgG4-Related Disease Presenting as a Submucosal Tumor of the Stomach Resected With Laparoscopic Endoscopic Cooperative Surgery: A Case Report. Surg Case Rep (2020) 6(1):93. doi: 10.1186/s40792-020-00851-8 32382972 PMC7206475

[19] Probst A Schaller T Sommer F Geissler B Agaimy A Messmann H. Immunoglobulin G4 (IgG4)-Related Disease of the Stomach - a Challenging Differential Diagnosis in Suspected Gastric Cancer. Z Gastroenterol (2019) 57(11):1298–303. doi: 10.1055/a-1013-4437 31739375

[20] Woo CG Yook JH Kim AY Kim J. IgG4-Related Disease Presented as a Mural Mass in the Stomach. J Pathol Transl Med (2016) 50(1):67–70. doi: 10.4132/jptm.2015.07.28 26420251 PMC4734962

[21] Seo HS Jung YJ Park CH Song KY Jung ES. IgG4-Related Disease in the Stomach Which Was Confused With Gastrointestinal Stromal Tumor (GIST): Two Case Reports and Review of the Literature. J Gastric Cancer (2018) 18(1):99–107. doi: 10.5230/jgc.2018.18.e8 29629225 PMC5881015

[22] Inoue K Okubo T Kato T Shimamura K Sugita T Kubota M. IgG4-Related Stomach Muscle Lesion With a Renal Pseudotumor and Multiple Renal Rim-Like Lesions: A Rare Manifestation of IgG4-Related Disease. Mod Rheumatol (2018) 28(1):188–92. doi: 10.3109/14397595.2015.1081743 26381653

[23] Cho MJ Moon HS Lee HS Park JH Kim JS Kang SH. Immunoglobulin G4-Related Disease in the Stomach Presenting as a Gastric Subepithelial Tumor: Case Report. Med (Baltimore) (2020) 99(36):e22078. doi: 10.1097/MD.0000000000022078 PMC7478664 32899079

[24] Berger Z Lea-Plaza Puig MI Varela C Becerra M Capetillo M Vargas J. [IgG4 Related Hypertrophic Gastropathy. Rep One Case] Rev Med Chil (2019) 147(1):119–24. doi: 10.4067/S0034-98872019000100119 30848775

[25] Bulanov D Arabadzhieva E Bonev S Yonkov A Kyoseva D Dikov T. A Rare Case of IgG4-Related Disease: A Gastric Mass, Associated With Regional Lymphadenopathy. BMC Surg (2016) 16(1):37. doi: 10.1186/s12893-016-0151-4 27255154 PMC4890503

[26] Muto O Tamakawa S Takahashi K Yokohama S Takasoe A Hirano F. IgG4-Related Disease Manifesting as Gastroduodenal Ulcer Diagnosed by an Endoscopic Biopsy. Intern Med (2020) 59(20):2491–7. doi: 10.2169/internalmedicine.4483-20.PMID: 32581158 PMC7662039 32581158

[27] Lim DY Cheng LT Tan DMY Al Jajeh I. Isolated IgG4-Related Gastric Disease Presenting as Diffuse Gastric Wall Thickening With Ulcer. J Radiol Case Rep (2018) 12(9):9–20. doi: 10.3941/jrcr.v12i9.3493 PMC6312045 30651919

[28] Bohlok A Khoury ME Tulelli B Verset L Zaarour A Demetter P. A Rare Presentation of IgG4 Related Disease as a Gastric Antral Lesion: Case Report and Review of the Literature. Int J Surg Case Rep (2018) 51:244–7. doi: 10.1016/j.ijscr.2018.08.065 PMC6138857 30218821

[29] Karagiannis P Gilbert AE Josephs DH Ali N Dodev T Saul L. IgG4 Subclass Antibodies Impair Antitumor Immunity in Melanoma. J Clin Invest (2013) 123(4):1457–74. doi: 10.1172/JCI65579 PMC3613918 23454746

[30] Wang H Xu Q Zhao C Zhu Z Zhu X Zhou J. An Immune Evasion Mechanism With IgG4 Playing an Essential Role in Cancer and Implication for Immunotherapy. J Immunother Cancer (2020) 8(2):e000661. doi: 10.1136/jitc-2020-000661 32819973 PMC7443307

